# Interview skills – psychiatry reel to reality

**DOI:** 10.1192/bjo.2021.783

**Published:** 2021-06-18

**Authors:** Mathuri Tharmapoopathy, Santosh Kumar, Abishan Thavarajah

**Affiliations:** 1 Newcastle University Medical school; 2 Roseberry Park Hopsital, Tees, Esk and Wear Valley NHS trust; 3 Queen Mary University and Barts and the London School of Medicine and Dentistry

## Abstract

**Aims:**

This reel analysis identifies quotes and actions of fictional characters from TV shows, namely: Hercules Poirot, Sherlock Holmes and House who can demonstrate learning points for clinical students to use within real psychiatric practice, using scientific theories such as the Hypothetico-deductive model, Empirical falsification and Occam's razor. This analysis explores what an ideal psychiatric interview consists of and what can be learnt from these characters and implemented within medical education.

**Method:**

Each show was watched by one researcher over the period of March to August 2020. The researcher noted insightful quotes which were relevant to one of the three philosophical theories. Quotes were included if they demonstrated deduction skills, revealed a character's ethos and supported the Calgary-Cambridge model of interviewing such as building rapport. 32 quotations were collected in total and narrowed to 6 quotations. These were then analysed, learning points were made and linked to the Calgary Cambridge model.

**Result:**

Dr House demonstrates objectivity when taking a patient's history. He utilises empirical falsification when diagnosing to avoid missing a differential diagnosis. Detective Poirot displays how empathic listening allows disclosure of details in the history, which would have otherwise been omitted. Additionally, he illustrates the importance of collateral interviewing which allows one to identify misinterpretations and inconsistencies. Sherlock teaches us the importance of perception regarding mismatching information which can help to gather new facts. All three characters interview beginning with open questions to more closed questions, supplementing with deductive reasoning in order to solve cases. Objectivity, empirical falsification, empathetic listening and deductive reasoning are the key skills displayed by these characters, that medical students can most use in their own practices.

**Conclusion:**

The perfect interview discovers new information through synchronised collaboration, whilst adhering to the Hypothetico-deductive model of thought. A combination of the Calgary-Cambridge model of interviewing and skillset of the TV characters should be considered for implementation in some aspects of psychiatric interviewing. Medical education can utilise these TV shows to teach students how to conduct history-taking.

